# Standardised neutralising antibody assays are needed for evaluating COVID-19 vaccines

**DOI:** 10.1016/j.ebiom.2021.103677

**Published:** 2021-11-03

**Authors:** Youchun Wang

**Affiliations:** Division of HIV/AIDS and Sex-transmitted Virus Vaccines, Institute for Biological Product Control, National Institutes for Food and Drug Control (NIFDC), No. 31 Huatuo Street, Daxing District, Beijing 102629, China

In this article of *EBioMedicine*, Viviana Simon and colleagues show that severe acute respiratory syndrome coronavirus 2 (SARS-CoV-2) mRNA vaccine remains effective against emerging SARS-CoV-2 variants of concern/interest in neutralisation and binding activities of serological tests [1]. This provides in vitro laboratory evidence of the continued efficacy of vaccines against emerging SARS-CoV-2 variants.

As the COVID-19 outbreak continued, mutations occurred in the genome of SARS-CoV-2 lead to the emergence of various variants. Amino acid variation in functional proteins such as spike protein leads to changes in viral properties, especially immunity. WHO has named and strongly alerted variants of concern (VOC) and variants of interest (VOI) based on the monitoring and tracking of changes in viral transmissibility, severity and/or immunity. The most effective way to control and end the COVID-19 pandemic is to get a safe and effective vaccination. Although a wide range of vaccines against COVID-19 are available, with more than 100 vaccine products in clinical development from a range of different platforms, almost all of them were developed based on the original strain. The emergence of highly transmitted and immunologically altered variants, such as Delta, Beta, has raised concerns about the effectiveness of current vaccines.

The findings presented in Viviana Simon group's work tested the neutralisation activity of 15 mRNA-1273 vaccine and 15 BNT162b2 vaccine immunized sera against seven SARS-CoV-2 isolates derived from New York. Neutralisation reduction was found to be stronger against Lambda subvariant, followed by Beta and Alpha+E484K. And in the receptor binding domain and spike binding assay, vaccine immunized sera showed little change in binding activities. There is growing evidence that despite a slight decline in the effectiveness of vaccines in preventing infection by variants, more than 90% effective in preventing severe COVID-19 disease and death has been observed [2]. A pair of recent in vitro experiments suggested that Beta strain may be the most resistant variant to vaccine protection [3,4], but it did not lead to vaccine failures [5].

Currently, the lack of uniform and standardized laboratory methods (neutralising antibody assay, binding antibody assay, cellular immune response assay) for evaluating the immune response of COVID-19 vaccination greatly limits the comparability of the protective effect of different vaccines against variants. The gold standard method for measuring vaccine-induced neutralizing antibodies is virus neutralisation test and plaque reduction neutralisation test using live viruses. However, virus strains isolated from different laboratories are geographically restricted and often limited in number, and may undergo laboratory variation during culture [6]. Although pseudovirus-based neutralisation assays have a high practical value as an alternative method, the vectors for constructing pesudovirus and testing procedures are different in different laboratories. In addition, ELISA-based assays for binding antibodies, due to the different antigens used by different laboratories, including full-length spike protein, RBD protein, S1 protein, S2 protein, again resulted in poor comparability between results. Thus, it is imperative to establish standardised antibody assays with international standards [7], recommended standard strains, clear cut-off values and others to make the result comparable. It is recommended to learn from the standardized assessments of neutralising antibodies for HIV/AIDS vaccine development of the Duke Central Reference Laboratory, and establish a reference laboratory to provide operating protocols, test samples, determination criteria, assay protocols, support documents, and verify the ability of neutralising antibody detection assays in different laboratories [8].

It is also particularly important to explore correlation between vaccine induced neutralising antibody and clinical protective effects. A pair of recent papers show that neutralising antibody levels are highly predictive of immune protection from symptomatic or serious SARS-CoV-2 infections [9,10]. However, the exact quality and quantity of neutralizing antibodies required to prevent SARS-Cov-2 infection in humans is unclear. With the standard neutralisation assays and international standard (7) or reference panel, the titer of neutralizing antibody against different variants could be evaluated and compared. As clinical data accumulate, correlation with viral protection and minimum protection levels of neutralising antibody against variants should be determined. It is worth mentioning that post-vaccine sera reference sample panel from the NIAID SARS-CoV-2 Assessment of Viral Evolution in vitro group initiative was used in Viviana Simon's paper.

With the establishment of natural immunity in the population, SARS-CoV-2 variants are still evolving and immune escape variants may emerge. The findings from Viviana Simon's lab provide additional basis for exploring the protective effects of vaccines against variants, yielding laboratory data that vaccines still provide excellent immune protection against variants. On this issue, the antigenicity changes of the current SARS-CoV-2 may not be sufficient to require replacement of the current vaccine strain. The updating and research on new SARS-CoV-2 vaccines should focus on those inducing higher titer of neutralizing antibodies by finding strong immunogens, varying the time between vaccine doses, heterologous prime-boost strategy, nasal vaccines, rather than changing virus strains periodically.

## Contributors

Youchun Wang read the commented paper, sorted out other related references and wrote this commentary.

## Declaration of Competing Interest

The author declares no conflict of interest.
